# A Random Forest Model for Post-Treatment Survival Prediction in Patients with Non-Squamous Cell Carcinoma of the Head and Neck

**DOI:** 10.3390/jcm12155015

**Published:** 2023-07-30

**Authors:** Xin Zhang, Guihong Liu, Xingchen Peng

**Affiliations:** 1 State Key Laboratory of Biotherapy and Cancer Center, West China Hospital, Sichuan University, Chengdu 610041, China; zhangxin13@wchscu.cn (X.Z.); liuguihong1987@scu.edu.cn (G.L.); 2 Department of Biotherapy, Cancer Center, West China Hospital, Sichuan University, Chengdu 610041, China

**Keywords:** head and neck, non-squamous cell carcinoma, machine learning, survival prediction

## Abstract

Background: Compared to squamous cell carcinoma, head and neck non-squamous cell carcinoma (HNnSCC) is rarer. Integrated survival prediction tools are lacking. Methods: 4458 patients of HNnSCC were collected from the SEER database. The endpoints were overall survivals (OSs) and disease-specific survivals (DSSs) of 3 and 5 years. Cases were stratified–randomly divided into the train & validation (70%) and test cohorts (30%). Tenfold cross validation was used in establishment of the model. The performance was evaluated with the test cohort by the receiver operating characteristic, calibration, and decision curves. Results: The prognostic factors found with multivariate analyses were used to establish the prediction model. The area under the curve (AUC) is 0.866 (95%CI: 0.844–0.888) for 3-year OS, 0.862 (95%CI: 0.842–0.882) for 5-year OS, 0.902 (95%CI: 0.888–0.916) for 3-year DSS, and 0.903 (95%CI: 0.881–0.925) for 5-year DSS. The net benefit of this model is greater than that of the traditional prediction methods. Among predictors, pathology, involved cervical nodes level, and tumor size are found contributing the most variance to the prediction. The model was then deployed online for easy use. Conclusions: The present study incorporated the clinical, pathological, and therapeutic features comprehensively and established a clinically effective survival prediction model for post-treatment HNnSCC patients.

## 1. Introduction

Head and neck cancer (HNC) is common, and there are about 932,000 new cases and 467,000 deaths worldwide each year, according to GLOBOCAN 2020 [1]. The incidence and mortality are the seventh highest compared to other cancer sites [1,2]. According to an epidemiological study based on the Surveillance, Epidemiology, and End Results (SEER) database, the incidence of HNC is still increasing, with an average annual percent change of 0.6% [3]. The histological type is mainly head and neck squamous cell carcinoma (HNSCC) derived from mucosal epithelium, accounting for over 90% [4]. HNSCC studies are relatively sufficient, thanks to which the 5-year survival for HNSCC has increased from 55% during 1992–1996 to 66% during 2002–2006, according to SEER [5]. Even many unique risk factors of HNSCC have been identified, such as Epstein–Barr virus (EBV), human papillomavirus (HPV), and areca nut, which are not common risk factors in other cancers, unlike tobacco or alcohol. Additionally, different with head and neck non-squamous cell carcinoma (HNnSCC), most HNSCCs (excluding those in the oral cavity) are treated with radiation therapy primarily [6]. Although there are enormous differences between HNSCC and HNnSCC, studies of HNnSCC are still very limited since it is uncommon and heterogeneous. Therefore, the present study aimed to provide more insight into HNnSCC.

Survival and recurrence evaluation is essential for patients and physicians, and can influence various clinical decisions or choices. However, there is still no appropriate survival prediction model for HNnSCC, especially an integrated tool for heterogenous primary sites and pathological subtypes. There are only a limited number of survival-prediction nomograms for squamous cell carcinoma [7,8,9], small-cell carcinoma [10], soft tissue sarcoma of the head and neck [11], and carcinoma of salivary glands [12,13]. The limitation of the nomogram is that it is the visualization of linear regression, which is easy to use, but not appropriate for some complex problems. Thus, to establish a survival prediction tool for HNnSCC with highly heterogeneous sites and pathological types, non-linear ensemble learning algorithms were utilized in this study, which is expected to have higher accuracy [14]. Meanwhile, the SEER database is also appropriate for filtering out sufficient data for uncommon HNnSCC, which is essential for machine learning algorithms.

Thus, this is the first study using large-scale data to develop a survival prediction tool of HNnSCC with the help of machine learning and the SEER database. The developed prediction model was also deployed on a website. Physicians worldwide can evaluate the survivals of HNnSCC using this website easily.

## 2. Methods

### 2.1. Data Source

This study is reported in accordance with the TRIPOD (Transparent Reporting of a Multivariable Prediction Model for Individual Prognosis or Diagnosis) statement.

**Figure 1:**
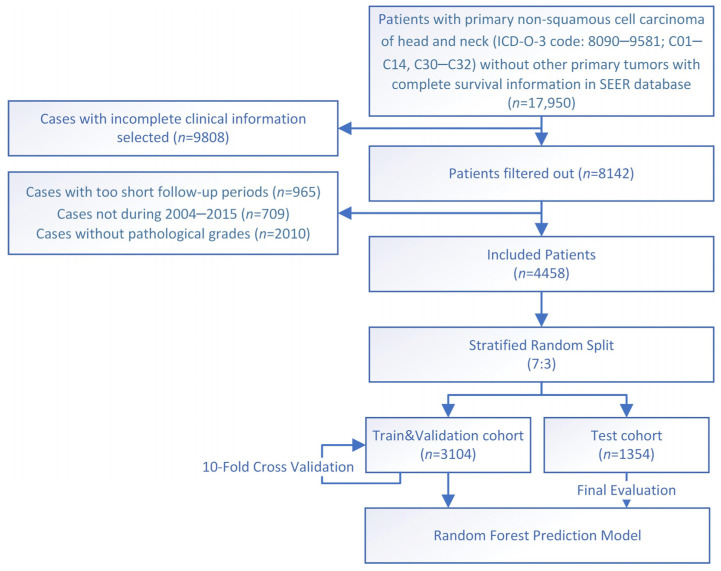
The flow chart for study identification, screening, and inclusion.

Data used in the present study are all from the SEER database. Data during 2000–2019 (17 registries) are downloaded. The International Classification of Diseases for Oncology, Third Edition (ICD-O-3), site codes C01-C14 and C30-C32 are used to filter out all head and neck cancer cases. Then, data with the histological codes 8000–8084 (not otherwise specified (NOS) and squamous neoplasm) and 9590–9992 (blood system-related cancers) are excluded. Exclusion criteria also include (1) inclusion of incomplete clinical or pathological data; (2) multiple primary tumors; (3) follow-up periods less than 6 months; (4) included years not during 2004–2015. Finally, 4458 patients are included in the present study (Figure 1).

### 2.2. Endpoint and Variables

There are two primary endpoints, overall survival (OS) and disease-specific survival (DSS, also called cancer-specific survival here). The variables of the SEER database, “Survival months”, “SEER cause-specific death classification”, and “Vital status recode”, are used to decide the endpoints.

Sex, age, race, marital status at diagnosis, living area, tumor sites, histopathological type and grade, TNM staging, tumor size, involved lymph nodes, and treatment information are included in the present study for analyses. Three subgroups of marital status are identified: married, never married, and others (widowed and divorced, mainly). The data about living area includes rural/urban, and median household income, which has been adjusted to 2019 according to inflation by the SEER database. Site-specific factors are converted to involved cervical lymph node regions. Treatment variables are whether chemotherapy, radiotherapy, or surgery was received. It is worth noting that all these treatment variables are collected after treatments, which means the analyses and prediction models in the present study can only be used after physicians’ treatment, rather than interfering with their judgement.

Site codes of ICD-O-3, C01-C06, and C14.8 (overlapping lesion of lip, oral cavity, and pharynx) are defined as the oral cavity. C07-C08 are defined as the salivary glands. C9-C10 (excluding C10.1 (anterior surface of the epiglottis)) and C14 (excluding C14.8) are defined as the oropharynx. C11 is defined as the nasopharynx. C30-C31 is defined as the nasal cavity and sinuses. C10.1, C12-C13, and C32 are defined as the larynx, glottis, and hypopharynx.

As for pathological types (also coding by ICD-O-3), “acinar cell neoplasms” (8550–8552), “adenomas and adenocarcinomas” (8140–8384), “complex mixed and stromal neoplasms” (8930–8991), “ductal and lobular neoplasms” (8500–8543), and “mucoepidermoid neoplasms” (8430) are analyzed separately. Cases of other pathological types are combined as “others” due to their rarity in collected data. Pathological grades are also collected from the SEER database, from Grade I (well differentiated) to IV (undifferentiated). 

### 2.3. Prediction Model Establishment

Nominal categorical variables are changed to dummy variables. Hazard ratios (HRs) of OS and DSS are separately calculated with Cox regression analysis. The statistically significant variables are input in the prediction model establishment. TNM staging is highly overlapped by variables of tumor size, involved lymph nodes, and distant metastases, and thus, not included in the regression analysis and establishment of the prediction model.

The random forest model is established with the “scikit-learn” package on Python. The patients are stratified–randomly divided into the train & validation cohort (70%) and test cohorts (30%). Tenfold cross validation are utilized in the process of choosing the most appropriate hyper-parameter values. Established models are exported as “*.pkl” files with the “joblib” package.

### 2.4. Prediction Model Evaluation

In the present study, the model performance is evaluated and exhibited through three types of curves. The receiver operating characteristic (ROC) curves are drawn to display the discrimination ability of the models. The area under curve (AUC) quantifies the results. Secondly, the calibration curves represent the concurrence between the actual survival and the predicted probability. The decision curve analysis (DCA) is used to compare the net benefit between the models and the traditional TNM-based linear prediction model, which is made to simulate traditional prediction methods based on TNM classification with “survival”, “rms”, and “nomogramEx” packages on R 4.2.2. Curves are plotted with Matplotlib. Performance on predicting 3-year OS, 3-year DSS, 5-year OS, and 5-year DSS are all evaluated with three types of curves. 

Although random forest, a decision tree-based machine learning method, can predict with higher accuracy than nomograms, it is not as intuitive as nomograms. To better understand the developed model, the Gini coefficients of the random forest model are shown in a heatmap. A higher Gini coefficient means that the factor is more important and contributes more information/variance to the developed model. 

### 2.5. Deployment

Considering the good evaluation results, an interactive website is established to improve the practical value of the present study. Entering clinical information required, 3-year OS, 3-year DSS, 5-year OS, and 5-year DSS can be calculated automatically. Django on Python is utilized for establishing the prediction website. 

### 2.6. Statistical Analysis

Categorical variables are described by both frequency and percentage. The 95% confidence interval (CI) of scaled variables is displayed. Kaplan–Meier analysis is performed for survival analyses. Cox regression analysis is performed for investigating prognostic factors and calculating HRs. The AUCs between models and the TNM-based Cox regression model are compared with Delong’s test. The *p* value is considered statistically significant when <0.05. The statistical analysis of the present study is performed mainly with SPSS Statistics 26.

## 3. Results

### 3.1. Characteristics and Regression Analysis

Characteristics of the train & validation cohort, test cohort, and comprehensive data are summarized in Table 1. A total of 4458 cases were included in the present study, divided into the train & validation cohort with 3104 cases and the test cohort with 1354 cases. The median age is 45–59, and 51.1% are female patients. In total, 56.6% of patients were married, and 22.8% were never-married patients. The most frequently involved site of HNnSCC is the salivary glands (67.4%), followed by the oral cavity (24.4%). The common pathological types include “adenomas and adenocarcinomas” (28.4%), “mucoepidermoid neoplasms” (44.4%), and “acinar cell neoplasms” (8.3%). A total of 42.8% cases were evaluated to be Grade II (moderately differentiation), which is more than other grades. When diagnosed, 39.8% of patients are Stage I. Overall, 21.4% are Stage II, and 14.5% are Stage III. In total, 48.3% of tumors are smaller than 20 mm, while 13.7% are larger than 40 mm. Most involved lymph nodes are Level II (12.3%), followed by Level I (8.9%) and Level III (6.5%). Referring to the treatments, 95.5% of patients received surgery. A total of 49.6% received radiotherapy, and 10.7% received chemotherapy.

HRs of OS were calculated and are listed in Table 2, and those of DSS are in Table 3. The results show that younger patients have better OS and DSS. Male (HR = 1.221–1.281) patients have shorter survival than female patients. Marital status at diagnosis has no significant effect on DSS, while married patients have a better OS than widowed/separated patients (HR = 1.291). Areas with lower household incomes tend to have poor OS and DSS (HR = 1.039–1.720; compared to the richest subgroup). The race and whether living in urban or rural areas shows no effect on either OS or DSS in multivariate analyses. Large tumor size (>20 mm, ≤40 mm HR = 1.625–1.818; >40 mm HR = 2.648–3.101) and Involved level I, II, and IV cervical lymph nodes (HR = 1.247–1.548) were associated with both poor OS and DSS, while Involved level III (HR = 1.429) was associated with only poor OS. Distant metastases also indicate poor OS and DSS (HR = 3.406–3.758). Compared to salivary gland tumors, tumors of the larynx/hypopharynx (HR = 2.209–2.608) had both poorer OS and DSS. The prognosis of tumors of the oral cavity, nasal cavity/paranasal sinus, nasopharynx, or oropharynx is similar to that of salivary gland tumors. In analyses of pathological subtypes, adenomas and adenocarcinomas (HR = 1.480–1.782) and acinar cell neoplasms (HR = 1.867, only significant for DSS) have a poorer prognosis than other subtypes. Pathological grades show significant association with both OS (HR = 1.589–3.836) and DSS (HR = 3.040–9.006). 

As for treatments, all surgery (HR = 0.668–0.675) and chemotherapy (HR = 1.212–1.271) both influence OS and DSS, while radiotherapy (HR = 1.343) only influences DSS. However, it is important to note that these treatment variables also included potential information about patients’ performance status and clinical stages of cancer, since early-stage patients with better performance status tend to receive surgery, and advanced patients need more chemotherapy and radiotherapy. Therefore, all these treatments variables should be collected and used for survival prediction or analyses after treatments. Survivals of different treatment combinations cannot be compared here and should not be used for treatment choice.

### 3.2. Model Evaluation

**Figure 2:**
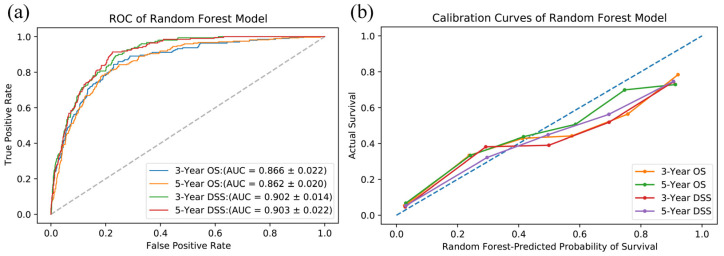
(**a**) ROC curves. AUCs of the test cohort range from 0.866 to 0.903, which shows a good discrimination ability of the model. (**b**) Calibration curves show consistency between predicted probability and actual survival of OS and DSS of 3 and 5 years.

Prediction models using random forest were established with tumor size, involved cervical lymph nodes, and distant metastases, instead of TNM classification, because of information overlapping. The performance of the prediction model was evaluated with the 1354 cases in the test cohort. The model performance and comparison with traditional TNM-based Cox regression methods are displayed in Table 4. The TNM-based Cox regression model is used to simulate traditional prediction methods. The AUCs of random forest range from 0.862 to 0.903, which are all significantly better (*p* < 0.001) than the regression prediction with TNM stages. The AUCs of random forest can also be seen graphically in Figure 2a.

**Figure 3:**
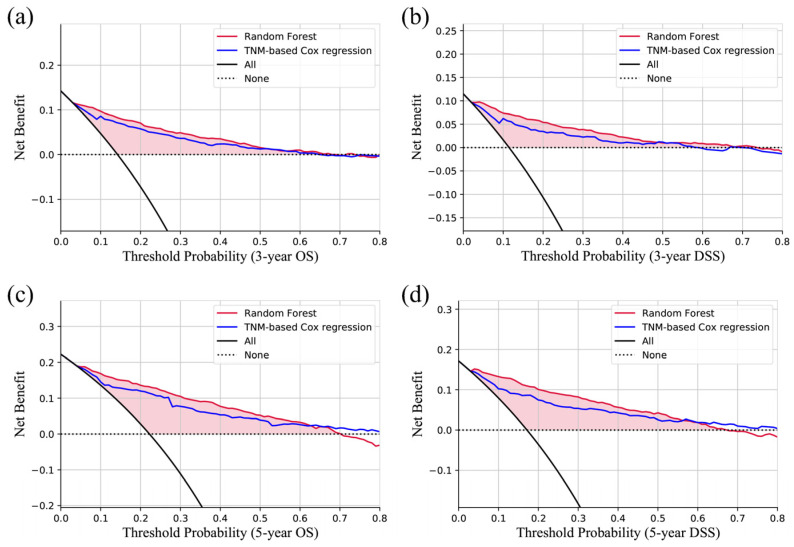
Decision curves of the test cohort show that the model is better than the TNM-based Cox regression model. (**a**) 3-year OS; (**b**) 3-year DSS; (**c**) 5-year OS; (**d**) 5-year DSS.

The calibration curves are all close to the diagonal (Figure 2b), which indicates that the predicted probabilities are similar to the actual proportions. The results of DCA (Figure 3) show that no matter whether predicting 3-year OS, 3-year DSS, 5-year OS, or 5-year DSS, the random forest models are all better than the traditional TNM-based regression model, which is to simulate traditional prediction methods. 

**Figure 4:**
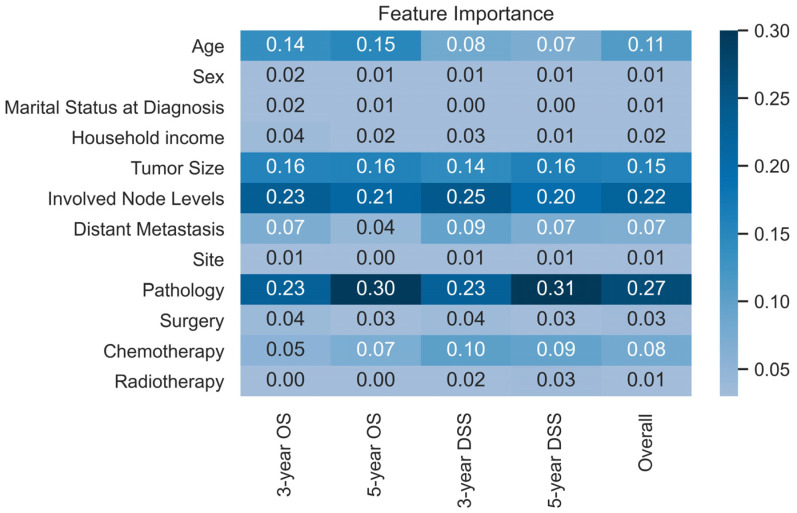
The heatmap of the feature importance (Gini coefficient) in the prediction model, from which the developed model can be better understood.

Figure 4 shows the importance of each variable (Gini coefficients) included in the prediction models. The pathology (including both type and grade), involved nodes levels, tumor size, and age have the most critical influence on survival prediction. Age is apparently more important in OS prediction than in DSS prediction. 

### 3.3. Prediction Website

**Figure 5:**
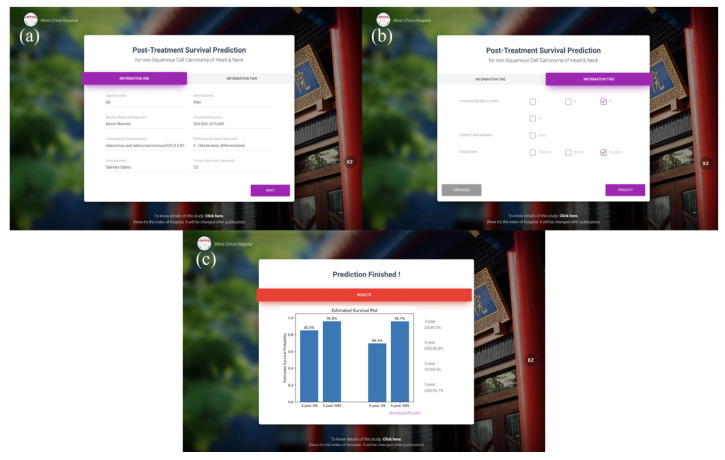
Interface and a result example of the prediction website (**a**), (**b**) The interfaces for information entering; (**c**) Predicted results of OS and DSS of 3 and 5 years.

An interactive website able to be easily used was deployed online. It can be accessed with “http://42.192.80.13:5001/ (accessed on 25 July 2023)”. With the needed variables filled, the predicted results can be computed automatically. Figure 5 shows examples of the online prediction tool.

## 4. Discussion

Although there are a larger number of HNC studies, the studies of HNnSCC are still limited and need to be improved because of its rarity and heterogeneity. Existing studies and many guidelines for head and neck mainly focus on HNSCC, and the prognosis prediction tool for HNnSCC is insufficient. Also, the high heterogeneity of HNnSCC indeed brings difficulties for establishing an integrated prediction tool. For example, the 5-year OS of small-cell carcinoma of the head and neck is 27% [15], while the 5-year OS of giant-cell sarcoma of the head and neck is 54.6% [16] and that of Ewing sarcoma of the head and neck could be 87% [17]. Traditional approaches, like nomograms based on linear fitting, might be inappropriate and not as accurate as machine learning. To date, no prediction tool can be used on heterogeneous HNnSCC and perform well. Thus, the present study is aimed at exploring predicting OS and DSS of HNnSCC with an ensemble learning algorithm and large-size clinical data.

In the present study, many variables were found to be prognosis predictors of OS or DSS in multivariate regression analysis (Table 2 and Table 3). Tumor size and involved levels of cervical lymph nodes replace T and N stages in the multivariate analysis of this study. Involved node levels I, II, and IV were found to be independent predictors of both OS and DSS, but the node level III was not found statistically significant in analyses of DSS. In many HNC studies, level IV is regarded as a prognostic factor [18,19], while there are studies mentioning levels I and III, but not as commonly as level IV [20,21]. However, the effect of the involved node levels on HNnSCC survival is still limited. Surgery and chemotherapy are significant for both of DSS and OS, while radiotherapy is only significant for DSS. Furthermore, radiotherapy and chemotherapy have higher HRs, which might be because they tend to be used in patients with more advanced stages. Meanwhile, the surgery choice made by surgeons must indicate earlier stages and better potential performance status. These also indicate that the treatment variables in the prediction of the present study can only be used after the treatment choice is decided. In comparison, many studies reported prognostic predictors of HNSCC. The role of age as a predictor of HNSCC prognosis has been controversial, with some studies considering it a predictor [22,23], while others not [24,25]. Other factors, such as marital status, primary site, tumor size, involved lymph nodes, distant metastasis, and treatments, can also be independent prognostic factors of OS in HNSCC [26], similar to the predictors found in this study on HNnSCC.

Many non-medical factors need to be acknowledged as potentially influencing the prognosis of tumors, including socioeconomic factors, marital status, and personal habits. According to available data of the SEER database, this study includes marital status at diagnosis and the economic level of the residence area. Among these variables, whether the residence is in rural or urban areas does not affect the prognosis, but the economic level does. Groups with a lower median household income tend to have worse prognoses. Similar results have also been mentioned in various studies [27]. However, there is also a study analyzing both the income and education level, reporting that survival is related to education rather than household income [28]. As for the marital status at diagnosis, the widowed/divorced/separated subgroup always tends to have a potentially higher HR than the married and never-married subgroups. This phenomenon has also been reported by other studies dividing marital status into three or more subgroups, which is possibly because of a large alteration of living status; more psychological stress; and the start of worse bad habits, like alcohol abuse [29,30,31,32]. Furthermore, according to the systematic review by Fugmann et al., the stability of the marital status of cancer patients is different from the general population [33], especially for younger patients [34]. Therefore, investigating the effect of divorce, as well as being widowed or separated, might be more important for tumor patients. Our exploration of socioeconomic and marital factors is preliminary and needs further studies to provide us with more insights.

Prognosis predictors were then input for the development of machine learning prediction models. Machine learning is the process in which the computation imitates humans to recognize the patterns of data. Machine learning algorithms have been utilized in many fields of clinical medicine [35], like disease diagnosis [36], survival prediction [36], and molecule screening [37]. The performance of a machine learning model is constantly improving with more sample cases input. Since HNnSCC is rare compared to HNSCC, only the databases with a considerable amount of data can have sufficient sample sizes. Thus, the present study chose the data from the SEER database because most databases contain no rare tumor data, such as The Cancer Genome Atlas (TCGA). Machine learning and the SEER database are the appropriate combination to explore things about HNnSCC. The random forest model is also selected among machine learning algorithms in the present study because it was reported that random forest is the best among 179 classifiers, which is evaluated with 121 real-world datasets [38]. And, this is also consistent with our experience.

The ROC, calibration, and decision curves (Figure 2 and Figure 3) used for evaluating the developed prediction models show that the performance is good. The AUCs range from 0.862 to 0.903. There are several nomograms of HNnSCC. For example, the nomogram for small-cell carcinoma has a 5-year-DSS AUC of 0.75 [10], and the results of nomograms for minor salivary gland carcinoma predicting 3-year or 5-year OS and DSS are 0.837–0.871 [13]. In comparison, the performance of the prediction model of this study is still good, and there are even tumors of various pathological types from various sites of the head and neck. It might be because of the merits of ensemble machine learning, rather than a linear fitting.

However, one problem of ensemble machine learning algorithms is that the models are not as intuitive as nomograms, thus, the importance of input variables in the models displayed in a heatmap (Figure 4). (The feature importance here is the Gini coefficients.) The higher Gini coefficients mean the enormous contribution of the variable to the prediction. The pathology (including both of type and grade), involved nodes levels, tumor size, and age are the most important variables in the prediction models, which means that they might not be the most correlated to survival, but provide the most information for the prediction. Distant metastasis, surgery, and chemotherapy are also important. In contrast, radiotherapy is found to be much less useful for prediction, also indicating the difference between HNSCC and HNnSCC. Although sites, sex, marital status at diagnosis, and household income are found to be prognosis predictors, the importance is less than the variables mentioned above. 

Another problem of the machine learning prediction models is that if there is no interactive interface, they will be difficult to use in daily work compared to nomograms. Thus, the models were deployed on the server and can be used through the website developed (http://42.192.80.13:5001/ (accessed on 25 July 2023)), which expanded the application value of the present study. An example of the interface and result can be seen in Figure 5. 

However, there are still limitations in the present study. Firstly, the data are from the SEER database, which means it is real-world population-based data, but retrospective, with unavoidable selection bias. Meanwhile, retrospective treatment variables also limit the usage of this prediction model to only the post-treatment situation because potential information is implied, such as performance status information implied in the surgery variable. Secondly, most variables of the SEER database were included in analyses of the present study, but there is no information about molecular or genetic test results, as well as habits, and more detailed information about socioeconomic and marital factors, which limited further exploration. Most cancer databases that contain genetic or expression data and smoking data, like TCGA, do not include rare tumors, like HNnSCC. If there are variables in the SEER database possible to screen out potential molecular or smoking-related predictors, the prediction model performance must be much better. Thirdly, all data used in the present study are from a single database. Data from diverse sources are needed to validate the generalization ability further.

Considering the above limitations, which cannot be addressed in the short term, we reiterate the urgent need for larger and more comprehensive databases, coupled with increasingly affordable technologies, such as genetic and transcriptomic sequencing, to improve the performance of existing prediction models. Additionally, we should focus more on rare tumors, as common tumors are often found in multiple databases with significant overlap, while rare tumors are equally important for patients. Such efforts will be critical for improving the accuracy of prediction and, ultimately, enhancing patient care for individuals with HNnSCC. We will continue to monitor progress in this field and update the existing prediction model if more data become available.

## 5. Conclusions

The present study incorporated clinical, pathological, and therapeutic features comprehensively and established a clinically effective survival prediction model for post-treatment HNnSCC patients.

## Figures and Tables

**Table 1:** Patient characteristics of train & validation, test, and total cohort.

Characteristic	Cohort, No. (%)					
	Train & Validation		Test		Total	
Age, y						
<45	895	28.8%	391	28.9%	1286	28.8%
45–59	884	28.5%	407	30.1%	1291	29.0%
60–74	884	28.5%	385	28.4%	1269	28.5%
>74	441	14.2%	171	12.6%	612	13.7%
Sex						
Male	1507	48.6%	672	49.6%	2179	48.9%
Female	1597	51.4%	682	50.4%	2279	51.1%
Marital Status at Diagnosis						
Married	1763	56.8%	760	56.1%	2523	56.6%
Never Married	702	22.6%	313	23.1%	1015	22.8%
Widowed/Separated/Others	639	20.6%	281	20.8%	920	20.6%
Race						
White	2370	76.4%	1484	109.6%	3854	86.5%
Black	369	11.9%	217	16.0%	586	13.1%
Asian	327	10.5%	202	14.9%	529	11.9%
Others	38	1.2%	28	2.1%	66	1.5%
Median household income						
USD 0~45,000	238	7.7%	93	6.9%	331	7.4%
USD 45,000~60,000	653	21.0%	296	21.9%	949	21.3%
USD 60,000~75,000	1287	41.5%	533	39.4%	1820	40.8%
USD 75,000+	926	29.8%	432	31.9%	1358	30.5%
Living Area						
Urban	2752	88.7%	1215	89.7%	3967	89.0%
Rural	352	11.3%	139	10.3%	491	11.0%
TNM stage						
I	1218	39.2%	558	41.2%	1776	39.8%
II	669	21.6%	284	21.0%	953	21.4%
III	467	15.0%	180	13.3%	647	14.5%
IV	750	24.2%	332	24.5%	1082	24.3%
T stage						
T0	2	0.1%	0	0.0%	2	0.0%
T1	1329	42.8%	600	44.3%	1929	43.3%
T2	813	26.2%	357	26.4%	1170	26.2%
T3	481	15.5%	189	14.0%	670	15.0%
T4	479	15.4%	208	15.4%	687	15.4%
N stage						
N0	2484	80.0%	1067	78.8%	3551	79.7%
N1	257	8.3%	116	8.6%	373	8.4%
N2	348	11.2%	165	12.2%	513	11.5%
N3	15	0.5%	6	0.4%	21	0.5%
M stage						
M0	3014	97.1%	1319	97.4%	4333	97.2%
M1	90	2.9%	35	2.6%	125	2.8%
Tumor site						
Salivary gland	2091	67.4%	912	67.4%	3003	67.4%
Oral cavity	747	24.1%	341	25.2%	1088	24.4%
Nasal cavity/paranasal sinus	168	5.4%	53	3.9%	221	5.0%
Larynx/hypopharynx	37	1.2%	13	1.0%	50	1.1%
Nasopharynx	27	0.9%	16	1.2%	43	1.0%
Oropharynx	34	1.1%	19	1.4%	53	1.2%
Histopathologic type						
Acinar cell neoplasms	252	8.1%	118	8.7%	370	8.3%
Adenomas and adenocarcinomas	908	29.3%	359	26.5%	1267	28.4%
Complex mixed and stromal neoplasms	173	5.6%	77	5.7%	250	5.6%
Ductal and lobular neoplasms	98	3.2%	43	3.2%	141	3.2%
Mucoepidermoid neoplasms	1363	43.9%	617	45.6%	1980	44.4%
Others	310	10.0%	140	10.3%	450	10.1%
Histopathologic Grade						
I (Well differentiated)	820	26.4%	355	26.2%	1175	26.4%
II (Moderately differentiated)	1335	43.0%	573	42.3%	1908	42.8%
III (Poorly differentiated)	565	18.2%	265	19.6%	830	18.6%
IV (Undifferentiated)	384	12.4%	161	11.9%	545	12.2%
Surgery						
Yes	2964	95.5%	1300	96.0%	4264	95.6%
No	140	4.5%	54	4.0%	194	4.4%
Radiotherapy						
Yes	1541	49.6%	680	50.2%	2221	49.8%
No evidence	1563	50.4%	674	49.8%	2237	50.2%
Chemotherapy						
Yes	331	10.7%	179	13.2%	510	11.4%
No evidence	2773	89.3%	1175	86.8%	3948	88.6%
Tumor size						
≤20 mm	1488	47.9%	664	49.0%	2152	48.3%
>20 mm, ≤40 mm	1195	38.5%	500	36.9%	1695	38.0%
>40 mm	421	13.6%	190	14.0%	611	13.7%
Involved lymph nodes						
Levels I	271	8.7%	121	8.9%	392	8.8%
Levels II	319	10.3%	166	12.3%	485	10.9%
Levels III	174	5.6%	88	6.5%	262	5.9%
Levels IV	91	2.9%	41	3.0%	132	3.0%
Levels V	83	2.7%	42	3.1%	125	2.8%
Levels Parotid	143	4.6%	51	3.8%	194	4.4%
Levels Others	46	1.5%	20	1.5%	66	1.5%

**Table 2:** Results of univariate and multivariate Cox regression analyses of OS. HRs with significance are also displayed.

Characteristic	Univariate Analysis	Multivariate Analysis	
	*p* Value	HR (95%CI)	*p* Values
Age, y	<0.001		<0.001
<45		Reference	
45–59		1.948 (1.508, 2.517)	<0.001
60–74		2.524 (1.964, 3.243)	<0.001
>74		6.698 (5.159, 8.694)	<0.001
Sex	<0.001		
Female		Reference	
Male		1.281 (1.108, 1.480)	0.001
Marital Status at Diagnosis	<0.001		0.010
Married		Reference	
Never Married		1.062 (0.964, 1.206)	0.113
Widowed/Separated/Others		1.291 (1.095, 1.521)	0.002
Race	<0.001		0.823
White		Reference	
Black		1.047 (0.830, 1.321)	0.698
Asian		1.036 (0.799, 1.342)	0.792
Others		1.474 (0.605, 3.596)	0.393
Median household income (adj to 2019)	<0.001		0.001
USD 0~45,000		1.720 (1.267, 2.334)	0.001
USD 45,000~60,000		1.138 (0.919, 1.408)	0.235
USD 60,000~75,000		1.272 (1.074, 1.507)	0.005
USD 75,000+		Reference	
Living in Rural Area	0.009	1.177 (0.923, 1.503)	0.189
Tumor size	<0.001		<0.001
≤20 mm		Reference	
>20 mm, ≤40 mm		1.625 (1.371, 1.927)	<0.001
>40 mm		2.648 (2.165, 3.238)	<0.001
Involved lymph nodes			
Levels I	<0.001	1.247 (1.029, 1.510)	0.024
Levels II	<0.001	1.322 (1.051, 1.664)	0.017
Levels III	<0.001	1.409 (1.048, 1.893)	0.023
Levels IV	<0.001	1.493 (1.038, 2.147)	0.031
Levels V	<0.001	1.384 (0.984, 1.946)	0.062
Levels Parotid	<0.001	1.016 (0.786, 1.312)	0.906
Levels Others	<0.001	1.046 (0.676, 1.618)	0.840
Distant Metastasis	<0.001	3.406 (2.589, 4.481)	<0.001
Tumor site	<0.001		0.016
Salivary gland		Reference	
Oral cavity		0.990 (0.812, 1.207)	0.923
Nasal cavity/paranasal sinus		1.084 (0.827, 1.419)	0.560
Larynx/hypopharynx		2.209 (1.419, 3.437)	<0.001
Nasopharynx		0.850 (0.466, 1.552)	0.598
Oropharynx		0.864 (0.480, 1.554)	0.625
Histopathologic type	<0.001		<0.001
Acinar cell neoplasms		1.322 (0.932.1.875)	0.118
Adenomas and adenocarcinomas		1.480 (1.246, 1.757)	<0.001
Complex mixed and stromal neoplasms		0.800 (0.599, 1.068)	0.130
Ductal and lobular neoplasms		1.167 (0.847, 1.607)	0.344
Mucoepidermoid neoplasms		Reference	
Others		1.520 (1.187, 1.946)	<0.001
Histopathologic Grade	<0.001		<0.001
I (Well differentiated)		Reference	
II (Moderately differentiated)		1.589 (1.246, 2.026)	<0.001
III (Poorly differentiated)		3.443 (2.655, 4.465)	<0.001
IV (Undifferentiated)		3.836 (2.932, 5.017)	<0.001
Treatment			
Surgery	<0.001	0.675 (0.519, 0.877)	0.003
Radiotherapy	<0.001	1.091 (0.929, 1.281)	0.287
Chemotherapy	<0.001	1.271 (1.049, 1.540)	0.014

**Table 3:** Results of univariate and multivariate Cox regression analyses of DSS. HRs with significance are also displayed.

Characteristic	Univariate Analysis	Multivariate Analysis	
	*p* Value	HR (95%CI)	*p* Values
Age, y	<0.001		<0.001
<45		Reference	
45–59		1.514 (1.137, 2.014)	0.004
60–74		1.636 (1.234, 2.170)	0.001
>74		2.705 (1.978, 3.699)	<0.001
Sex	<0.001		
Female		Reference	
Male		1.221 (1.018, 1.464)	0.031
Marital Status at Diagnosis	0.011		0.187
Married		Reference	
Never Married		0.861 (0.668, 1.108)	0.244
Widowed/Separated/Others		1.137 (0.919, 1.406)	0.236
Race	0.008		0.525
White		Reference	
Black		1.107 (0.827, 1.482)	0.492
Asian		1.148 (0.839, 1.572)	0.389
Others		1.754 (0.642, 4.795)	0.273
Median household income (adj to 2019)	<0.001		0.015
USD 0~45,000		1.570 (1.084, 2.274)	0.017
USD 45,000~60,000		1.039 (0.796, 1.355)	0.779
USD 60,000~75,000		1.275 (1.033, 1.573)	0.024
USD 75,000+		Reference	
Living in Rural Area	0.016	1.143 (0.846, 1.544)	0.384
Tumor size	<0.001		<0.001
≤20 mm		Reference	
>20 mm, ≤40 mm		1.818 (1.450, 2.281)	<0.001
>40 mm		3.101 (2.408, 3.994)	<0.001
Involved lymph nodes			
Levels I	<0.001	1.548 (1.252, 1.914)	<0.001
Levels II	<0.001	1.353 (1.044, 1.753)	0.021
Levels III	<0.001	1.341 (0.963, 1.867)	0.083
Levels IV	<0.001	1.530 (1.059, 2.211)	0.024
Levels V	<0.001	1.392 (0.970, 1.998)	0.073
Levels Parotid	<0.001	1.185 (0.895, 1.569)	0.238
Levels Others	<0.001	0.843 (0.516, 1.377)	0.495
Distant Metastasis	<0.001	3.758 (2.792, 5.057)	<0.001
Tumor site	<0.001		0.001
Salivary gland		Reference	
Oral cavity		1.113 (0.862, 1.438)	0.412
Nasal cavity/paranasal sinus		1.143 (0.823, 1.587)	0.425
Larynx/hypopharynx		2.608 (1.603, 4.245)	<0.001
Nasopharynx		0.751 (0.364, 1.548)	0.437
Oropharynx		0.852 (0.420, 1.730)	0.658
Histopathologic type	<0.001		<0.001
Acinar cell neoplasms		1.867 (1.218, 2.863)	0.004
Adenomas and adenocarcinomas		1.782 (1.432, 2.219)	<0.001
Complex mixed and stromal neoplasms		0.858 (0.602, 1.223)	0.398
Ductal and lobular neoplasms		1.228 (0.846, 1.783)	0.279
Mucoepidermoid neoplasms		Reference	
Others		1.414 (1.003, 1.993)	0.048
Histopathologic Grade	<0.001		<0.001
I (Well differentiated)		Reference	
II (Moderately differentiated)		3.040 (1.990, 4.644)	<0.001
III (Poorly differentiated)		8.372 (5.440, 12.885)	<0.001
IV (Undifferentiated)		9.006 (5.818, 13.940)	<0.001
Treatment			
Surgery	<0.001	0.668 (0.493, 0.906)	0.010
Radiotherapy	<0.001	1.343 (1.085, 1.662)	0.007
Chemotherapy	<0.001	1.212 (0.976, 1.505)	0.014

**Table 4:** Comparison of performance on test data between developed random forest model and TNM classification; *p* values are calculated by Delong’s test.

Survival	Test Cohort		
	AUC	95%CI	*p* Value
OS-3 year			<0.001
Random Forest	0.866	0.844–0.888	
TNM-based Cox	0.831	0.802–0.860	
OS-5 year			<0.001
Random Forest	0.862	0.842–0.882	
TNM-based Cox	0.836	0.808–0.864	
DSS-3 year			<0.001
Random Forest	0.902	0.888–0.916	
TNM-based Cox	0.861	0.825–0.897	
DSS-5 year			<0.001
Random Forest	0.903	0.881–0.925	
TNM-based Cox	0.872	0.846–0.902	

## Data Availability

The data underlying this article are available in the SEER Database: Incidence-SEER Research Plus Data, 17 Registries, Nov 2021 Sub (2000–2019). Data can be accessed with the Surveillance Research Program, National Cancer Institute SEER*Stat software (version 8.4.1) (seer.cancer.gov/seerstat (accessed on 6 September 2022)).
